# Case of Sudden Death after Anesthetic in Latent Case of Shell Gas Poisoning

**Published:** 1919-01

**Authors:** 


**A Case of Sudden Death after an Anesthetic in a Latent Case of Shell Gas Poisoning.** By PHILIP TURNER, Major, R. A. M. C. *The Lancet*, September 21, 1918.

The fatal accident related below is most unusual, but the possibility of sudden death occurring after an anesthetic, in a man who has been exposed to the action of poison gases, should be borne in mind, and special care taken in similar cases.

The patient was suffering from a wound in the right foot received on the previous day. General condition was good; no symptoms of gas poisoning. Chloroform was administered and a shell fragment removed from the wound. Very little anesthetic was required. Pulse and color were normal throughout the operation. When the patient was about to be removed from the operation room, it was noticed that he had suddenly become slightly cyanosed; but rapidly his color appeared quite good again and his respiration normal. He was practically out of anesthesia and even sat up and spat out a little mucus. About three minutes later, however, he died. There had been no vomiting, and no obstruction could be felt on digital examination of the pharynx. Post-mortem examination showed the whole heart very dilated, especially the right auricle and ventricle. There was no obstruction of any sort in the air passages. Tracheitis was present and the lungs were slightly congested. The whole appearance was characteristic of gas poisoning and the amount of cardiac dilatation suggested that the gas was phosgene. Death was due to primary cardiac failure. The patient probably made some slight muscular effort, which his weakened and dilated heart was unable to bear.

After an anesthetic, all movements should be most severely checked in patients where there is the slightest suspicion of gas poisoning.

